# Determinants of mortality in trauma patients following massive blood transfusion

**DOI:** 10.4103/0974-2700.76839

**Published:** 2011

**Authors:** Kanchana Rangarajan, Arulselvi Subramanian, Ravindra Mohan Pandey

**Affiliations:** Laboratory Medicine & Blood Bank, Jai Prakash Narayan Apex Trauma Centre, AIIMS, New Delhi, India; 1 Department of Biostatistics, AIIMS, New Delhi, India

**Keywords:** FFP:PRBC ratio, massive blood transfusion, mortality, outcome, trauma

## Abstract

**Aim:**

This study was designed to find out the factors influencing mortality in trauma patients receiving massive blood transfusion (MBT).

**Materials and Methods:**

Records of all patients admitted during December 2007 to November 2008 at a Level I Trauma Center emergency and who underwent massive transfusion (≥10 units of packed red cells in 24 h) were retrospectively analyzed. Death during the hospital stay was considered as the study outcome and various demographic, laboratory, and clinical parameters were included as its potential determinants.

**Statistical Analysis:**

Bivariate and multivariate logistic regression analyses were done to identify the risk factors associated with mortality.

**Results:**

Of the 4054 transfused patients who were admitted to the trauma center during the study period, 71 (1.8%) patients underwent massive transfusion. Of this, there were 37 survivors and 34 nonsurvivors (48%). The median overall ISS was 27 (22–34). The patients who died had shorter mean length of hospital stay, shorter mean duration of intensive care unit (ICU) stay, and low admission Glasgow Coma Scale (GCS) compared to the survivors (*P* < 0.01). The mean prothrombin time (PT) and the mean activated partial thromboplastin time was significantly high (*P* < 0.01) among nonsurvivors. Total leukocyte count (TLC ≥ 10,000 cells/cubic mm), GCS ≤ 8, the presence of coagulopathy and major vascular surgery were the four independent determinants of mortality in multivariate logistic regression analysis. The FFP:PRBC (fresh frozen plasma:packed red cells) ratio and PC:PRBC (platelet concentrate:packed red cells) ratio calculated in our study was not statistically significant in correlation to the in hospital mortality.

**Conclusions:**

Overall mortality among the MBT patients was comparable with the studies in the literature. Mortality is not affected by the amount of packed red cells given in the first 12 h and the total number of packed red cells transfused. Prospective studies are required to further validate the determinants of mortality and establish guidelines for MBT.

## INTRODUCTION

Blood transfusion is an essential component of trauma services. Severely injured trauma victims most often undergo massive blood transfusion (MBT) due to extensive damage and blood loss. Massive transfusion is life saving in many instances and at times imposes a serious threat on the treating physicians, given the consequences and complications of massive transfusion.[1–5] They may also cause an acute depletion of blood bank resources. MBT may cause hypocalcemia, hyperkalemia, hypomagnesemia, acid–base disturbances, and hypothermia as a complication.[1–5] The dilutional effects of MBT without appropriate coagulation factor replacement include thrombocytopenia and coagulopathy. Other than these, citrate toxicity and transfusion-associated acute lung injury have also been reported.[6,7] Recent studies indicate that packed red cells (PRBC) replacement with an adequate and appropriate use of fresh frozen plasma (FFP) and platelet concentrates (PC) early in trauma management prevents dilutional effects and markedly improve the coagulopathic bleeding in trauma patients.[8–10] Various MBT protocols have been developed to standardize the blood transfusion management of trauma patients.[11–15] Recent reports suggest an improved survival following MBT.[16,17] Appropriate use of blood components, prompt correction of coagulopathy, decreased operative times for the initial emergency operation, more aggressive and efficient use of rewarming procedures are some of the measures which have significantly decreased mortality.[4,16–22]

Current retrospective studies suggest a higher FFP to packed red cell ratio to be associated with a significant decrease in the mortality rate.[5,23–25] Although there is no uniform consensus on the optimal ratio which decides this survival advantage, there is a tendency for researchers to include the ratio of FFP to packed red cells as 1:1 in the massive transfusion guidelines worldwide.[11–15,26–28] The study was designed to evaluate the factors influencing mortality (immediate outcome) in trauma patients receiving massive transfusion and explore its determinants.

## MATERIALS AND METHODS

Records of 4054 patients admitted in the trauma center emergency who underwent transfusion during a 1 year period (December 2007 to November 2008) were screened. Seventy one patients who had an MBT episode and had complete data set for analysis were separated and analyzed. MBT was defined as infusion of 10 units or more of packed red cells in 24 h. Patients with pre-existing coagulation disorders were excluded from the study. The variables analyzed include patients’ demographics, length of stay in hospital and in ICU, mode of injury (blunt or penetrating), injury severity score (ISS), Glasgow Coma Scale (GCS), organs injured, admission laboratory parameters, FFP:PRBC ratio, PC:PRBC ratio and the outcome being dead or alive.

### Data analysis

The data were extracted on a predesigned proforma and managed on an excel spread sheet. All the entries were checked for any possible data entry error. Quantitative variables were summarized as mean ± SD for normally distributed and median (min–max) for nonnormally distributed variables. Comparisons between the dead and alive patients were done using Student’s *t*-test for mean values of continuous variables and *χ*^2^ or Fisher exact test for categorical variables. To know the risk factors of death among trauma patients with MBT, analysis was done in two steps: (1) Bivariate logistic regression analysis was done with each variable to compute unadjusted odds ratio (95% CI) and (2) Variables found statistically significant at *P* = 0.10, was considered as candidate risk factors and all such factors were simultaneously considered in the stepwise multivariate logistic regression to know the independent strength of association of the factors with the outcome (death). STATA 10.0 Statistical Software was used for data analysis. All the statistical tests used were two sided. In this study, *P* < 0.05 was considered as statistically significant.

## RESULTS

Of the total 4054 transfused patients, there were 71 cases (1.8%) of MBT. The mean age of the study group was 34 ± 12.9 years (mean ± SD); 37 were from surgery, 12 from orthopedics, and 22 from neurosurgery departments. There were six females. Forty-six (65%) patients sustained motor vehicle accidents, 17 (24%) had injury due to interpersonal violence, 6 (8.5%) had injury due to fall, and 2 (2.8%) had occupational injury. Majority of the injuries were blunt (64), and only seven patients suffered penetrating injuries. The median length of stay in hospital was 12 (5–28) days. The median overall ISS was 27 (22–34). There were 37 survivors and 34 nonsurvivors. The overall mortality was 48%. The maximum number of PRBC units transfused in an MBT patient during the hospital stay was 26 units in one patient and 24 units in another patient, both of whom survived. The total number of blood components consumed for MBT were 1025 packed red blood cells, 1007 FFP, and 601 PC.

The demographic and injury profile of patients at admission is shown in Table 1. Age distribution was statistically comparable between the survivors and non survivors. The patients who died had shorter mean length of hospital stay, shorter median duration of intensive care unit (ICU) stay, and low median Glasgow Coma Scale (GCS upon admission) compared to the survivors (*P* < 0.01). The mean prothrombin time (PT) and the mean activated partial thromboplastin time (APTT) were significantly high (*P* < 0.01) among nonsurvivors. All the admitted MBT patients underwent emergency surgery and the intraoperative variables were also included in the analysis. Vascular surgery encompasses the repair of blood vessels throughout the body with the exception usually of those within the cranium and those intrinsic to the heart. Surgery involving repair of major vessels such as popliteal, femoral, and brachial arteries were considered as major vascular surgeries in this study.

**Table 1:** Demographic profile of patients at admission

Variables	Outcome	
	Dead (*n* = 34)	Alive (*n* = 37)
Age (mean ± SD)	35.3 ± 14.9	32.7 ± 10.7
Male sex (%)	32 (94.1)	33 (89.2)
Length of stay in days*	5 (2–12)	22 (11–36)
Period of ICU stay in days*	5 (2–12)	15 (8–25)
Type of trauma (%)		
Motor vehicle accident	21 (61.7)	25 (69.4)
Fall	3 (8.8)	3 (8.3)
Interpersonal violence	10 (29.4)	7 (19.4)
Occupational accidents	0 (0)	2 (2.8)
Mode of injury (%)		
Blunt	31 (88.2)	33 (89.2)
Penetrating	4 (11.8)	3 (8.1)
ISS	27 (25–32)	29 (20–34)
GCS*	12.5 (7–15)	15 (13–15)
Anatomic area involved (%)		
Head	15 (44.1)	10 (27)
Major vessels	9 (26.5)	6 (16.2)
Liver	7 (21.2)	6 (16.2)
Spleen	2 (5.8)	6 (16.2)
Intestine	2 (5.8)	7 (18.9)
Kidney	2 (5.8)	0 (0)
Pelvis	5 (14.7)	5 (13.5)
Chest	5 (14.7)	5 (13.5)

* *P* < 0.01;

OTHER VARIABLES NOT SIGNIFICANT; VALUES IN THE TABLE ARE EXPRESSED AS MEAN ± SD, OR NUMBER (%) OR MEDIAN (MIN–MAX).

The bivariate analysis of patients’ initial clinical features, admission laboratory parameters, type of surgery, and intraoperative variables are shown in Table 2. The nonsurvivors had low hemoglobin (≤8 g/dL, *P* < 0.01), high total leukocyte count (TLC) ≥10,000 × 10^3^ /cubic mm, *P* < 0.01), low GCS (GCS ≤ 8, *P* < 0.01), the presence of hypovolemic shock [defined as >40% blood loss (American College of Surgeons Committee on Trauma, Advanced Trauma Life Support Program (1989)) diagnosed by: tachycardia, hypotension, cold clammy skin, decreased loss of consciousness, low urine output] on admission (*P* < 0.02), the presence of coagulopathy (PT ≥ 20 and APTT ≥ 50, *P* < 0.01) and underwent major vascular surgery (*P* < 0.04) compared to survivors. Low platelet count (<100 × 10^3^ cells/cumm) was not found to be significant between the survivors and nonsurvivors. The FFP:PRBC and the PC:PRBC ratio calculated were not found to be significant and did not predict mortality in our study.

**Table 2:** Risk factors of death among trauma patients with massive blood transfusion: results of bivariate logistic regression analysis

Variables	Outcome		*P* value
	Dead (*n* = 34)	Alive (*n* = 37)	
Low hemoglobin ≤ 8 (g/dL)	10 (29.4)	2 (5.4)	0.01
Low platelet count (<100 × 10^3^ cells/cumm)	20 (58.8)	18 (48.6)	0.18
Sodium (mEq/L)			0.20
≥145	12 (35.3)	4 (10.8)	0.06
≤135	10 (29.4)	15 (40.5)	
Potassium (mEq/L)			0.27
≥5	4 (11.7)	1 (2.7)	
≤3.5	20 (58.8)	21 (56.8)	
TLC (cells/cumm)			0.01
≤4000	5 (15.1)	1 (2.7)	
≥10,000	16 (48.5)	6 (16.2)	
ISS ≥ 25	27 (79.4)	25 (67.6)	0.30
Admission GCS ≤ 8	27(72.9)	15(44.1)	0.01
Presence of hypovolemic shock on admission	27 (79.4)	19 (51.4)	0.02
Presence of coagulopathy	22 (64.7)	4 (10.8)	0.01
PRBC transfused > 20 units	7 (20.6)	3 (8.1)	0.17
PRBC transfused in first 12 h	19 (55.9)	16 (43.2)	0.28
Type of surgery			0.14
Exploratory laparotomy	11 (32.4)	17 (45.9)	
Craniotomy	13(38.2)	8(21.6)	
Vascular and orthopedic surgery	10(29.3)	12(32.4)	
Intraoperative variables			
Intraoperative use of ionotropes	26 (83.9)	22 (59.5)	0.03
Systemic BP < 90 mmHg	19 (59.4)	13 (35.1)	0.05
Major vascular surgery	5 (15.6)	1 (2.7)	0.04
Presence of severe acidosis	1 (3.2)	0 (0)	0.45
Presence of prolonged hypotension	5 (15.6)	0 (0)	0.02
FFP:PRBC ratio (>0.8)	17 (50)	15 (40.5)	0.47
PC:PRBC ratio (>0.6)	15 (44.1)	12 (32.4)	0.33

**Figure 1:**
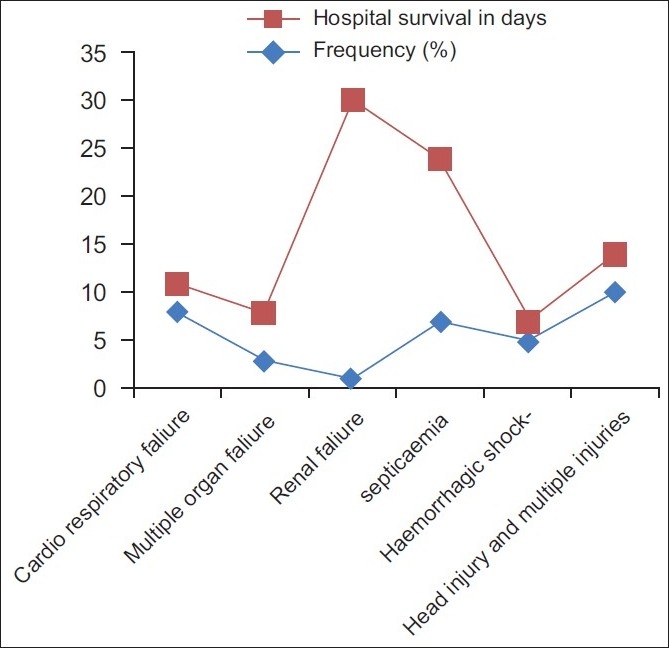
Causes of death in massively transfused trauma patients with frequency (%) and Length of Survival (days)

**Figure 2:**
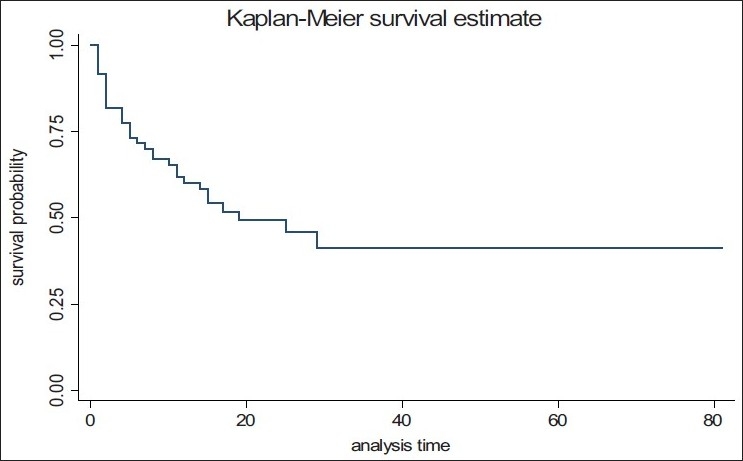
Kaplan-Meir survival analysis in massively transfused trauma patients

The factors which were significant in bivariate analysis were entered in to multivariate logistic regression analysis. In stepwise multivariate logistic regression analysis, following factors were found to be independent significant factors: high TLC [OR (95% CI): 25.9 (0.6–1053.5)], low GCS [OR (95% CI): 11.2 (1.3–96.1)], the presence of coagulopathy [OR (95% CI): 80.9 (7.9–822.6)], and major vascular surgery [OR (95% CI): 29.1 (1.1–741.5)] [Table 3]. Mortality was not associated with the amount of PRBC transfused in the first 12 h. Figure 1 lists the causes of death in the 34 nonsurvivors. The most common cause of death was closed head injury with associated multiple injuries including orthopedic fractures, blunt chest, and abdominal trauma. Death due to hemorrhage and subsequent shock was seen in five patients mainly due to the inability to correct coagulopathy in these patients. The early causes of death were closed head injury, hemorrhagic shock, multiple organ failure, and cardiorespiratory failure. The late causes of death include septicemia and renal failure. The Kaplan–Meier survival analysis curve is shown in Figure 2. The median survival time during the hospital stay was 19 days.

**Table 3:** Results of the final multivariate analysis–odds ratio of the significant variables

Variables	Unadjusted odds ratio	Adjusted odds ratio
Low hemoglobin ≤ 8 (g/dL)	0.3 (0.02–3.8)	–
TLC ≥ 10,000 cells/cumm	6.6 (2.1–21.1)	25.9 (0.6–1053.5)
GCS ≤ 8	7.29 (1.5–36.3)	11.2 (1.3–96.1)
Presence of hypovolemic shock on admission	3.65 (1.3–10.5)	–
Presence of coagulopathy	15.12 (4.3–52.9)	80.94 (7.9–822.6)
Major vascular surgery	6.66 (0.73–60.4)	29.1 (1.1–741.5)

## DISCUSSION

This study discusses the MBT practice at a level I trauma center with mortality as the primary endpoint. It lays emphasis on the factors associated with higher mortality of severely injured trauma victims. It also highlights the fact that the volume of PRBC transfused within the first 12 h as well as total units transfused does not affect the immediate outcome of trauma victims.

Comparison of studies on MBT is limited by the variability in the definitions used by different authors.[16,18,19,29] MBT has been defined by some authors as replacement of one entire blood volume within 24 h, as replacement of 50% of the total blood volume in 3 h or as transfusion of 20 units of packed red blood cells.[1,16,18,19] Vaslef *et al*. considered massive transfusion as 50 units of blood products in 24 h.[18] In the outcome analysis done by Cinat *et al*., MBT was analyzed on those who received greater than 50 units of packed red blood cells or whole blood in the 48 h following admission to the emergency department.[16]

We defined MBT as infusion of 10 units or more of packed red cells in 24 h. There are varied reports in the literature on the incidence of MBT.[17,20,29] Como *et al*. reported an incidence of 2.6%. Malone *et al*. reported 2.7% while Huber-Wagner *et al*. reported an overall 13%.[17,20,21] Our incidence of MBT was 1.8% which was in the lower end of the spectrum.

We derived four independent risk factors of mortality: (1) TLC ≥ 10,000; (2) GCS ≤ 8; (3) the presence of coagulopathy; and (4) major vascular surgery. There are few studies in the literature analyzing the possible risk factors affecting the mortality of the MBT victims. Harvey *et al*. did a retrospective analysis of 43 patients including 20 trauma victims and identified severe coagulopathy to be a common phenomenon after MBT.[22] Cosgriff *et al*. showed in their prospective analysis of 58 patients four significant risk factors namely pH < 7.1, temperature < 34°C, injury severity score > 25, and systolic blood pressure < 70 mmHg to be independent variables defining mortality.[4] They also showed that the conditional probability of developing coagulopathy was 98% when all the four-risk factors were present. In their 4-year retrospective review of 141 MBT patients, Velmahos *et al*. identified three independent intraoperative variables, need for aortic clamping, use of inotropes and intraoperative time with a systolic blood pressure of 90 mmHg or less to be associated with mortality.[19]

In a study by Cinat *et al*., analysis of 45 patients of MBT revealed male sex, major vascular injury, high injury severity score, severe acidosis, prolonged hypotension, refractory hypothermia, and decreased use of platelet transfusion to be associated with poor outcome in the study group.[16] Vaslef *et al*. did multiple logistic regression analysis on 44 trauma patients in which base deficit >12 was the only significant factor which emerged as the independent risk factor of mortality.[18] Mitra *et al*. showed injury severity score, initial coagulopathy measured by APTT, and the presence of head injuries to be the independent predictors of mortality.[29] Huber-Wagner *et al*. demonstrated variables such as age over 55 years, GCS ≤ 8, MBT ≥ 20 units of PRBC, thromboplastin time < 50%, and injury severity score ≥ 24 to be high risk factors for mortality.[17] Finally, McLaughlin *et al*. recognized four independent risk factors: heart rate > 105 bpm, systolic blood pressure < 110 mmHg, pH < 7.25 and hematocrit < 32%, and created an algorithm to analyze the risk of MBT.[30]

Coagulopathy was found to be one of the significant independent predictors of mortality in our study with highest odds ratio (*P* < 0.01). We found an incidence of 65% coagulopathy in our study. Cinat *et al*. reported an incidence of 78% in nonsurvivors in their study group. Aggressive correction of coagulopathy in the latter part of their study improved the survival following MBT.[16] In one of the large studies in literature on MBT, Huber-Wagner *et al*. found high odds ratio for developing coagulopathy for increasing volumes of blood transfusion.[17] Harvey *et al*. demonstrated coagulopathy rate of 44% in 43 patients subsequent to MBT.[22] In another study by Mitra *et al*. coagulopathy was found to be an independent multivariate clinical feature associated with increased mortality among MBT victims. Further, coagulopathy was noted at admission in these patients demonstrating the need for early recognition and correction of this defect in these patients.[29] Based on this, some authors have suggested early, aggressive correction of this coagulopathy using a 1:1 ratio of plasma to red blood cell units.[11–15,26–28]

Our study revealed a low GCS (≤8) in 73% of nonsurvivors and was found to independently predict mortality. Low GCS has been analyzed by some authors as a risk factor for mortality.[18,21] GCS ≤ 8 was illustrated to be the second strongest predictor of mortality in a large study enrolling 1062 MBT victims (odds ratio = 4.6).[17]

High TLC was found to be an independent predictor of mortality. There were 48.5% of nonsurvivors who had a high TLC compared to 16.2% of survivors. Among those who were dead, only five patients had leukopenia. There was only one study in the literature analyzing the survival rate, leukopenia and acidosis in MBT recipients. Hakala *et al*. did a study on 23 MBT victims exceeding 50 units of red cells or whole blood. Their study demonstrated leukopenia (for 5 days) to be a regular phenomenon in all the patients with a mortality of 30%.[31] It must, however, be noted that five of the 23 patients had transfusion of more than 100 units of blood in their study while the maximum number of PRBC units transfused in any single patient in our study was 26 units.

The association of major vascular surgery with higher mortality has not been reported in the literature. Among them, 15.6% of the nonsurvivors who underwent major vascular surgery had higher mortality rates [OR (95% CI): 29.1 (1.1–741.5)] compared to the survivors (2.7%). Other intraoperative variables though significant in univariate analysis did not emerge as significant variables in multivariate analysis. It should be noted that the volume of PRBC transfused in the first 12 h and the total amount of PRBC transfused were not significant predictors of mortality in our study group. Although earlier studies have shown a direct relationship between mortality and the amount of PRBC transfused, we did not derive a significant relationship between the two.[21,29,32,33] Many recent retrospective studies suggest the ratio of FFP:PRBC:PC ratio as 1:1:1 to be optimal and to improve mortality[5,23–25] Although many researchers agree upon the survival benefit obtained due to the early, aggressive institution of plasma and platelets in massive transfusion victims, some question the mortality benefit of this high FFP:PRBC ratio. While some attribute this difference to mortality bias and effect of temporal factors, few others call for more prospective and randomized controlled trials before conclusions on optimal ratio is arrived at.[34–36] The FFP:PRBC (>0.8) and the PC:PRBC ratios (>0.6) calculated in our study were not statistically significant in correlation with the in hospital mortality. Survival rates have been reported to be improving in the last decade mainly because of more aggressive correction of coagulopathy, efficient rewarming procedures, damage control surgery, and improvised blood banking procedures.[4,16–22] A brief review of the mortality rates in MBT rates was done.[4] Cinat *et al*. in their comparative analysis of the survival rates (early and late period of 5 years) demonstrated that mortality rates have significantly declined from 84% in the early period to 55% in the later period.[16] Vaslef *et al*. reported a higher rate of 57% in 44 patients of MBT defined as infusion of more than 50 units of blood.[18] Como *et al*. noted 39% mortality in a cohort of 147 injured patients receiving more than 10 units of RBCs.[20] In the study by Hakala *et al*., we derived a mortality rate of 30% among the 23 patients studied (MBT greater than 50 units of RBCs).[31] Malone *et al*. derived an overall mortality rate of 22% in a large study group of 1703 trauma patients including 421 MBT victims.[21]

## CONCLUSIONS

This study has reviewed the practice of MBT at a Level I trauma center. Overall mortality among the MBT patients was comparable with the studies in the literature. The study supports the existing literature that mortality is affected by coagulopathy and low GCS in a massively transfused victim. The amount of PRBC transfused does not affect the mortality of MBT patients. Also, the FFP:PRBC (>0.8) and the PC:PRBC ratios (>0.6) calculated in our study were not statistically significant in correlation to the in hospital mortality. Hence, appropriate transfusion therapy in the emergency may improve outcome and prevent deaths due to coagulopathy in a MBT setting. There is a need to study further the effect of TLC and major vascular surgery in MBT patients in a larger study population. More prospective studies are required to study risk factors and establish guidelines for MBT.
